# The ideal site of cement application in cement augmented sacroiliac screw fixation: the biomechanical perspective

**DOI:** 10.1007/s00068-022-02187-4

**Published:** 2022-12-12

**Authors:** Christoph Emanuel Albers, Ivan Zderic, Philipp Kastner, Boyko Gueorguiev, Theodoros Herkules Tosounidis, Marius Johann Baptist Keel, Johannes Dominik Bastian

**Affiliations:** 1 Department of Orthopaedic Surgery and Traumatology, Inselspital, Bern University Hospital, University of Bern, Bern, Switzerland; 2 AO Research Institute Davos, Davos, Switzerland; 3 Department of Orthopaedic Surgery, Medical School, University of Crete, 71500 Heraklion, Crete Greece; 4 Trauma Center Hirslanden, Clinik Hirslanden Zurich, Witellikerstrasse 40, 8032 Zurich, Switzerland

**Keywords:** Fragility fracture, Pelvis, Sacroiliac screw, Cement augmentation, Biomechanical, Sacrum

## Abstract

**Purpose:**

To compare construct stability of cement augmented sacroiliac screws using two different cementation sites in a biomechanical fragility fracture model of the pelvis.

**Methods:**

A fracture model with an incomplete fracture of the sacral ala and complete fracture of the anterior pelvic ring mimicking a FFP IIB fragility fracture of the pelvis was established in five fresh frozen human cadaveric pelvises. Sacral fracture stabilization was achieved with bilateral 7.3 mm fully threaded sacroiliac screws. Cement augmentation was performed at the tip of the screw (body of S1; Group A) on one side, and at the midshaft of the screw (sacral ala; Group B) on the contralateral side. Biomechanical testing was conducted separately on both sides comprising cyclic loading of axial forces transferred through the tested hemipelvis from L5 to the ipsilateral acetabulum. Combined angular displacement in flexion and internal rotation (“gap angle”), angular displacement of the ilium in relation to the screw (“screw tilt ilium”), and screw tip cutout were evaluated.

**Results:**

Relative interfragmentary movements were associated with significantly higher values in group A versus group B for “gap angle” (2.4° vs. 1.4°; *p* < 0.001), and for “screw tilt ilium” (3.3° vs. 1.4°; *p* < 0.001), respectively. No significant difference was indicated for screw tip cutout between the two groups (0.6 mm [Group A] vs. 0.8 mm [Group B]; *p* = 0.376).

**Conclusion:**

The present study demonstrated less fragment and screw displacements in a FFP IIB fracture model under physiologic cyclic loading by cement augmentation of sacroiliac screws at the level of the lateral mass compared to the center of vertebral body of S1.

## Introduction

In older adults fractures of the pelvis are mainly caused by physiological loads during activities of daily living, induced by the patient’s own body weight or by low-energy trauma, in case of an extreme reduction in bone mass (e.g. severe osteoporosis) [1–4]. Moreover, older people are at risk for simple falls from standing positions: Thirty percent of the  people over 65 years of age and 50% of those over 80 years of age fall at least once each year, and older adults who fall once are likely to fall again within one year [5]. A simple fall can result in a fragility fracture of the pelvis (FFP) [3].

Once osteoporotic pelvic fractures have occurred, the destabilized pelvis causes a sudden onset of immobilizing pain. The loss of mobility determines the length of hospital stay and the discharge type; patients with pelvic fractures are representative for long hospitalization periods [6]. The amount of independently living patients decreased from 89% (before hospital admission) to 64% (at the point of discharge) leading to a loss of autonomy [7]. Finally, pelvic fractures in older adults with increased mortality rates are reported to range from 13 to 27% one year after the injury [8–11]. In summary, these fractures are indubitably severe injuries in older adults.

**Fig. 1:**
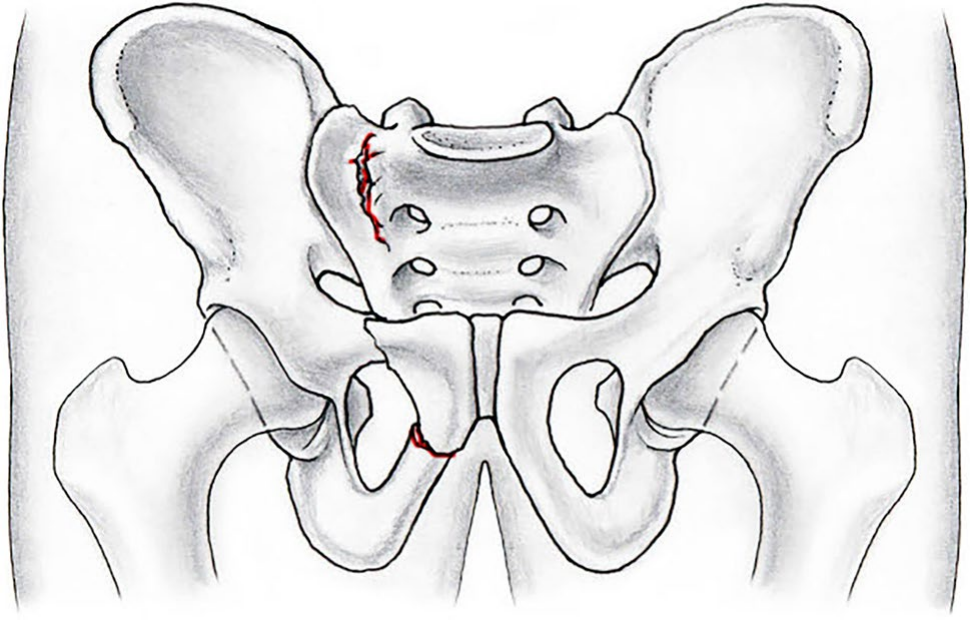
Schematic drawing with anteroposterior view of the pelvis. Internal rotation of the right hemipelvis caused pubic and ischial rami fractures anteriorly and an incomplete sacral fracture posteriorly (FFP IIB lesion according to Rommens et al. [3])

Decision making for treatment is guided by the inability of the pelvic ring to withstand physiological loads without displacement as determined by the degree of instability of various fracture types [3, 12]. The majority of FFPs present moderate instability caused by a non-displaced fracture of the posterior pelvic ring with or without anterior ring injuries (FFP Type II; e.g. FFP IIB, Fig. 1). In these injuries, the use of percutaneously placed sacroiliac screws with low access morbidity has been recommended for fixation [3]. However, a diminished screw purchase of sacroiliac screws as a monocortical screw device in cancellous, osteoporotic bone is a concern [13, 14]. Therefore, cement augmentation of sacroiliac screws at the level of the body of S1 vertebra was proposed to increase the anchorage between bone and implant and was a matter of preclinical research [15–20]. However, neither an advantage with regard to cyclic loading, nor a distinct clinical benefit of sacroiliac screw augmentation was evident in recent systematic reviews [21, 22] whereas shortened length of hospital stay and a reduced risk for general complications was noted in sacroiliac screws augmented with bone cement [23]. In contrast, the lateral sacral mass was identified clearly as the weakest part of the sacrum previously in cadavers of older adults [4, 24]. Accordingly, the sacral lateral mass serves as an optimal site for cement augmentation of sacroiliac screws. However, to our knowledge no study assessed this site for cement augmentation of sacroiliac screws. Thus, we hypothesize that cement augmentation of sacroiliac screws facilitated at the weakest part (lateral mass of S1) enhances construct stability compared to current practice (body of S1) and previous research. The aim of this study was to compare two different sites for cement augmentation of sacroiliac screws in their fixation strength in a biomechanical pelvic fracture model exposed to cyclic loading.

### Ethical approval

All pelvises were from donors who bequeathed their corpses to Science Care (Phoenix, AZ, USA) for use in medical science during their lifetime. Written consent is available. No local or national ethical approval was necessary. All experiments were carried out under the relevant guidelines and regulations.

## Methods

### Specimen and experimental protocol

Fresh-frozen ( – 20 °C) human cadaveric pelvic specimens (*n* = 5, three female; age 82 ± 9 years (mean  ± standard deviation), range 75–98 years) of donors with no history of skeletal disease were considered for biomechanical testing. Computed tomography (CT) scans (Revolution EVO, GE Healthcare, Chicago, IL, USA) were used to rule out any differences between specimens in (i) volumetric bone mineral density (BMD) of the sacrum at the level of S1 by a phantom (European Forearm Phantom QRM-BDC/6, QRM GmbH, Möhrendorf, Germany), (ii) presence of pre-existing deformations, fractures, neoplasms or in (iii) degenerative changes in the sacroiliac joint (as previously published [25]).

In all specimens, both sides of the sacrum were used to directly compare two operative techniques in the same bone quality. The left and right anatomical sites of each pelvis were randomly assigned in alternating manner for sacroiliac screw instrumentation with additional bone cement augmentation of either the screw tip (body of S1; group A), or the midshaft region (lateral mass of S1; group B). The left hemipelvis of each specimen was instrumented and tested first, keeping the contralateral right side intact. A total of three specimens were assigned for screw tip augmentation (group A), whereas two specimens were assigned for midshaft augmentation of their left hemipelvis (group B). After biomechanical testing of the left hemipelvis, the procedures were repeated for contralateral right sides after instrumentation with a sacroiliac screw including the complementary augmentation technique. Consequently, each group consisted of five specimens.

### Preparation of specimen and fracture model

Prior to preparation and biomechanical testing, the specimens were thawed at room temperature for 24 h. The soft tissue was completely dissected leaving only the bony pelvic structures with anterior and posterior sacroiliac, sacrospinosus, sacrotuberosus, and iliolumbar ligaments intact. Whereas the lumbar spine was dissected between L4 and L5 leaving L5 in continuity with the pelvis, the femora were completely removed. To generate a FFP IIB fracture with an “anterior ring disruption and posterior undisplaced sacral crush injury” a stable vertical paraforaminal sacral fracture was created in the lateral mass of the sacrum (zone 1 according to Denis classification [26]) anteriorly with an oscillating saw under direct visual and fluoroscopic control. Only the anterior cortex of the sacral ala at the level of S1 and S2 was sawed to a depth of approximately 25 mm whereas lesions to the sacroiliac joint and neuroforaminal area (zone 2) were avoided. Additionally, the iliolumbar and posterior sacroiliac ligaments were preserved. The symphysis was widely (20 mm) cut to discontinue the force transmission to the contralateral hemipelvis site through the anterior pelvic ring during load application (mimicking the “anterior ring disruption”).

### Screw modifications and instrumentation

Fully threaded self-tapping stainless steel 7.3 mm cannulated sacro-iliac (SI) screws (DePuy Synthes, Zuchwil, Switzerland) were used in this study, prepared for augmentation by means of two perforations measuring 1.6 mm in diameter and having an angular offset of 180° to each other. The perforations were custom drilled either at 7 mm and 12 mm distance from the screw tip for the screw tip augmentation (group A), or at 35 mm and 40 mm distance from the screw head for the midshaft augmentation (group B). The site for the perforations were defined based on preoperative CT scan measurements to identify the distance of the external cortex of the ilium at the screw entry point and the sacral lateral mass, which was found consistent and independent from individual specimens. The screw length was selected individually from these scans for each site separately. Due to the bi-lateral test design, care was taken that the final position of the screw tip did not cross the midline to avoid screw interdigitation or cement transgression to the contralateral side during the second test of the right, contralateral side.

**Fig. 2:**
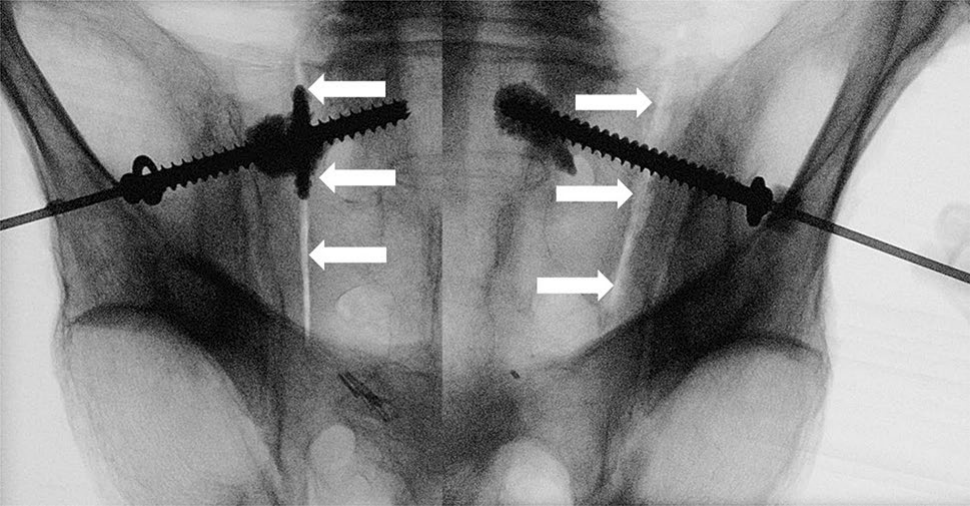
Images of the fluoroscan in outlet projection showing an instrumented specimen following screw tip augmentation (group A) on its left, and screw midshaft augmentation (group B) on its right anatomical site to demonstrate the instrumentation (white arrows: sacral fracture as created using an oscillating saw)

Sacroiliac screws were placed using the standard technique previously reported in detail [27, 28]. In addition, using the CT scan, individual entry and aiming points were pre-determined using a protocol as published previously [29]. Each cadaver was placed in prone position on a radiolucent operating table. Instrumentation started with insertion of a 2.8 mm guide wire under fluoroscopic control in the corridor of S1 in accordance with the described standard technique [30]. Subsequently, the lateral cortex was overdrilled with a cannulated 5.0 mm drill bit. The modified sacroiliac screw (of either group A or group B) with a standard washer was inserted and tightened over the guide wire. After removal of the guide wire, screw augmentation was performed using TRAUMACEM V + (DePuy Synthes, Zuchwil, Switzerland) bone cement kit and a modified side-opening cannula (12G, Unimed S.A., Lausanne, Switzerland) for each hemipelvis site separately. The cannula was pre-filled with cement and inserted through the cannulation of the screw. Then, two to three milliliters of bone cement were injected into the cancellous bone surrounding the screw. Using the side opening cannula, cement flow was directed sideways through the perforations, avoiding cement transgression through the screw cannulation over the S1 midline. Cement distribution was checked fluoroscopically and the injected amount was documented (Fig. 2). To ensure a minimal radiation dose, the image intensifier was used according to the ALARA (as low as reasonably achievable) principle. The same surgeon (experienced in cement augmentation techniques) performed all instrumentations. The cadavers were assessed for potential cement leakage into the fracture gap, neuroforamina, or spinal canal by intraoperative fluoroscopic imaging, and a post-operative CT scan.

### Biomechanical testing

**Fig. 3:**
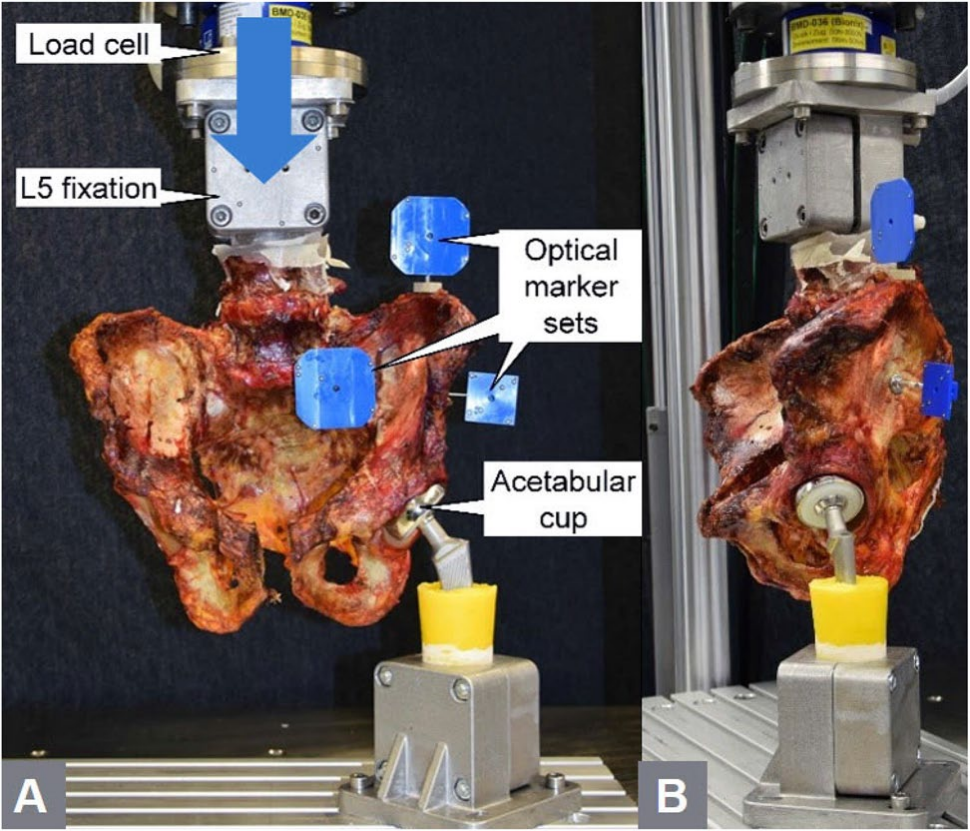
Photographs showing the cadaveric specimen and the biomechanical setup in **a** frontal (blue arrow: loading direction) and **b** lateral direction for testing in a one legged stance position under cyclic loading

The L5 vertebral body was embedded in polymethylmethacrylate (PMMA, SCS-Beracryl D-28, Swiss Composite, Jägestorf, Switzerland) cylindrical form, which served as machine anchorage point. The bone-PMMA fixation was enhanced via three 5.0 mm screws. Finally, optical marker sets were attached to the ilium, the sacrum, and the screw for optical motion tracking. Biomechanical testing was performed on a servo-hydraulic material testing system (MTS 858 Bionix, MTS Systems Corp., Eden Prairie, MN, USA) equipped with a 5 kN load cell. For that purpose, the specimens were oriented and mounted to the machine in simulated upright standing position (Fig. 3). The L5 embedding was firmly constrained to the machine transducer via a custom fixation. Axial forces initiated by the machine transducer were transferred through the tested hemipelvis site to the ipsilateral acetabulum, which was seated on a hip stem with attached hemiarthroplasty component, the former being firmly constrained to the machine base. The loading protocol started with a quasi-static ramp from 20 N preload to 100 N at a rate of 8 N/s, followed by axial cyclic loading under a physiologic loading profile [31] at 2 Hz until failure. During the cyclic test, the valley load was kept constant at 20 N, whereas the peak load, starting at 100 N, was monotonically increased at 0.006 N/cycle using an externally generated signal. The test was interrupted as soon as the machine transducer reached an axial displacement of 70 mm with respect to its position prior to test begin, which was sufficient to provoke construct failure.

### Data acquisition and parameters

**Fig. 4:**
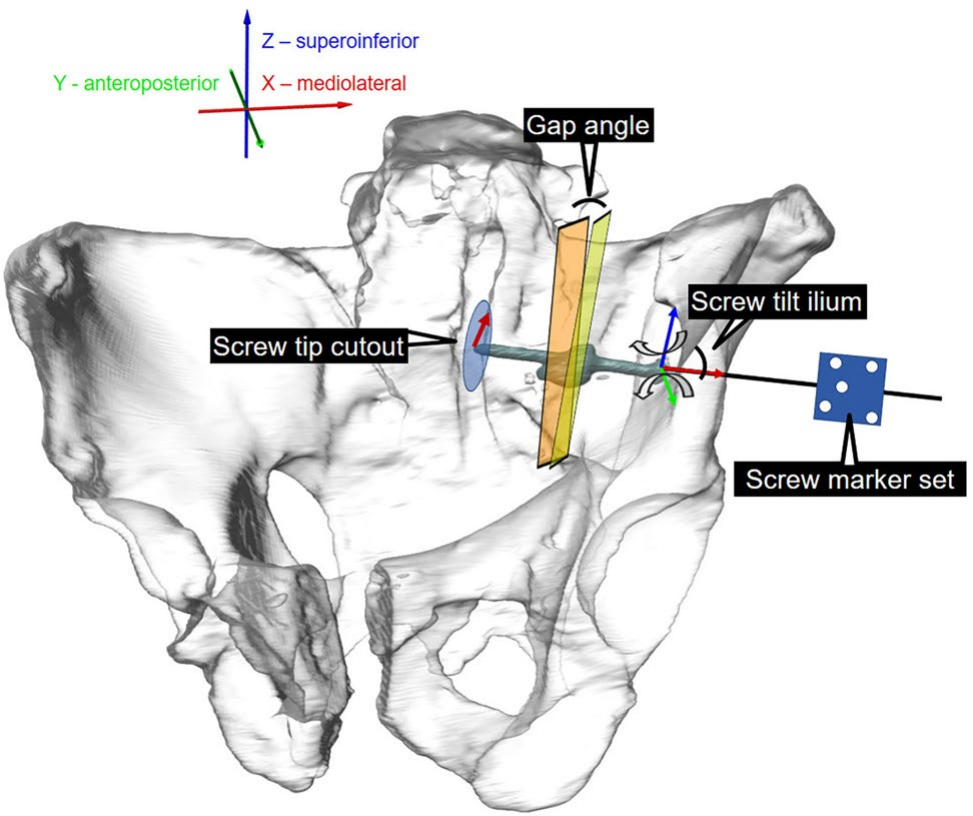
Visualisation of the biomechanical setup illustrating the motion tracking and the assessed parameters: (i) “Gap angle” is the flexural rotation of the medial sacral fragment relative to the lateral fragment with the gap opening between the two initially reduced osteotomy surfaces adjoining each other in the fracture gap, (ii) “Screw tilt ilium” is the movement of the screw relative to the ilium calculated in terms of its angular displacement in the coronal and transverse plane and (iii) “Screw tip cutout”is the screw tip displacement in those two planes

Machine data in terms of axial displacement (mm) and axial load (N) were recorded from the controllers at 128 Hz. Initial stiffness was calculated from the ascending slope of the load–displacement curve of the quasistatic test ramp within a load range 40–80 N. Two optical cameras (Aramis SRX, Carl Zeiss GOM Metrology GmbH, Braunschweig, Germany) continuously recorded the marker positions at 20 Hz for motion tracking, operating at resolution of 12 megapixel and maximum acceptance error of 0.004 mm [32]. Based on the motion tracking data, the following parameters were calculated: (i) the combined angular displacement in sagittal and coronal plane of the medial sacral fragment relative to the lateral fragment with the gap opening between the two initially reduced osteotomy/fracture surfaces adjoining each other in the fracture gap (“gap angle”), (ii) the movement of the screw relative to the ilium was calculated in terms of its angular displacement in the coronal and transverse plane (screw tilt ilium) and (iii) the screw tip displacement in those two planes (“screw tip cutout”) as shown in Fig. 4. All evaluations were performed under peak axial compression loading at five time points of cyclic testing after 10,000, 20,000, 30,000, 40,000 and 50,000 cycles. The latter cyclic number represented the highest rounded number when none of the specimens had failed yet. Fluoroscan images were taken in antero-posterior direction at the beginning of the cyclic test, and then every 500 cycles using a triggered C-arm (ARCADIS Varic, Siemens Healthineers AG, Erlangen, Germany) for inspection of the gradual decay of the specimens over time. For that purpose, the machine loading was interrupted at the corresponding peak load for two seconds.

### Statistical analysis

Statistical analysis among the parameters of interest was performed using SPSS software (v.27, IBM SPSS, Armonk, NY, USA). Mean and standard error of the mean (SEM) were calculated for each parameter of interest. The outcome measures were pooled over the five investigated time points for each parameter of interest and treatment group separately and reported in terms of median values and 95% confidence intervals (CI). Based on these pooled data sets, significant differences between the two treatments were assessed with Wilcoxon Signed-Rank tests, accounting for non-parametric data distribution. In addition, the reproducibility of each technique was assessed by pooling the SEM's of all investigated outcome measures—gap angle, screw tilt ilium, and screw tip cutout—for each group separately, and by statistically comparing them with the Wilcoxon Signed-Rank test. All p values < 0.05 were considered significant.

## Results

Morphometrically, BMD in the massa lateralis was homogeneously distributed between the sites having the screw tip (90.7 ± 12.5 mgHA/cm^3^) or the midshaft (81.1 ± 15.6 mgHA/cm^3^) augmented, *p* = 0.267. Screw augmentation was associated with cement leakage into the first neuroforamen following screw tip augmentation (group A) in one case and in all cases in the fracture gap but not into the true pelvis in group B. The initial axial construct stiffness was comparable for screw tip (37.1 ± 7.7 N/mm) or midshaft (39.5 ± 7.1 N/mm) augmentation, *p* = 0.729.

**Fig. 5:**
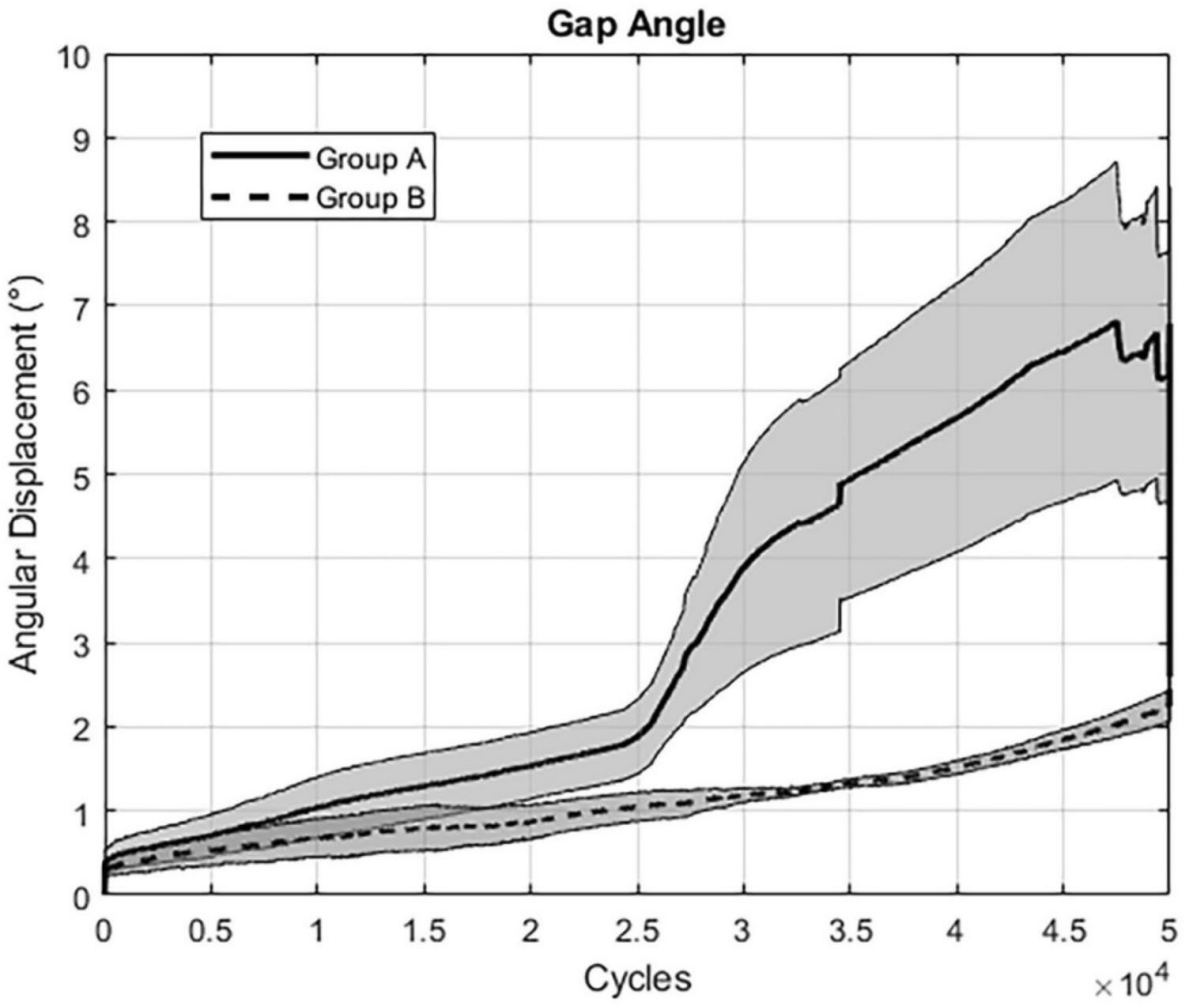
Graph showing results for the assessed parameter “Gap Angle”: The extent of angular displacement (in °) over the course of the first 50.000 cycles is depicted for both groups separately (mean ± SEM)

**Fig. 6:**
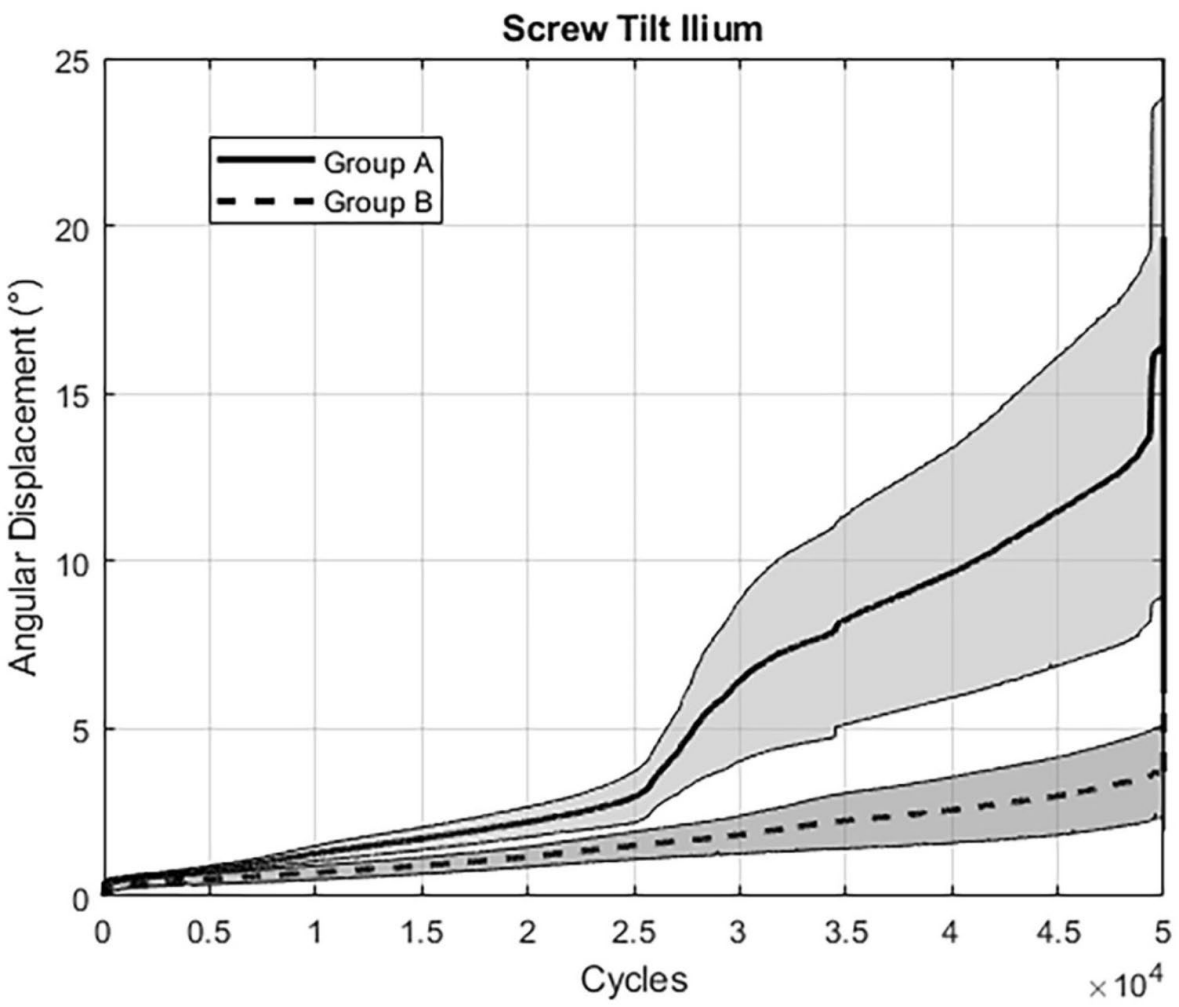
Graph showing results for the assessed parameter “Screw Tilt Ilium”: The extent of angular displacement (in °) over the course of the first 50.000 cycles is depicted for both groups separately (mean ± SEM)

**Fig. 7:**
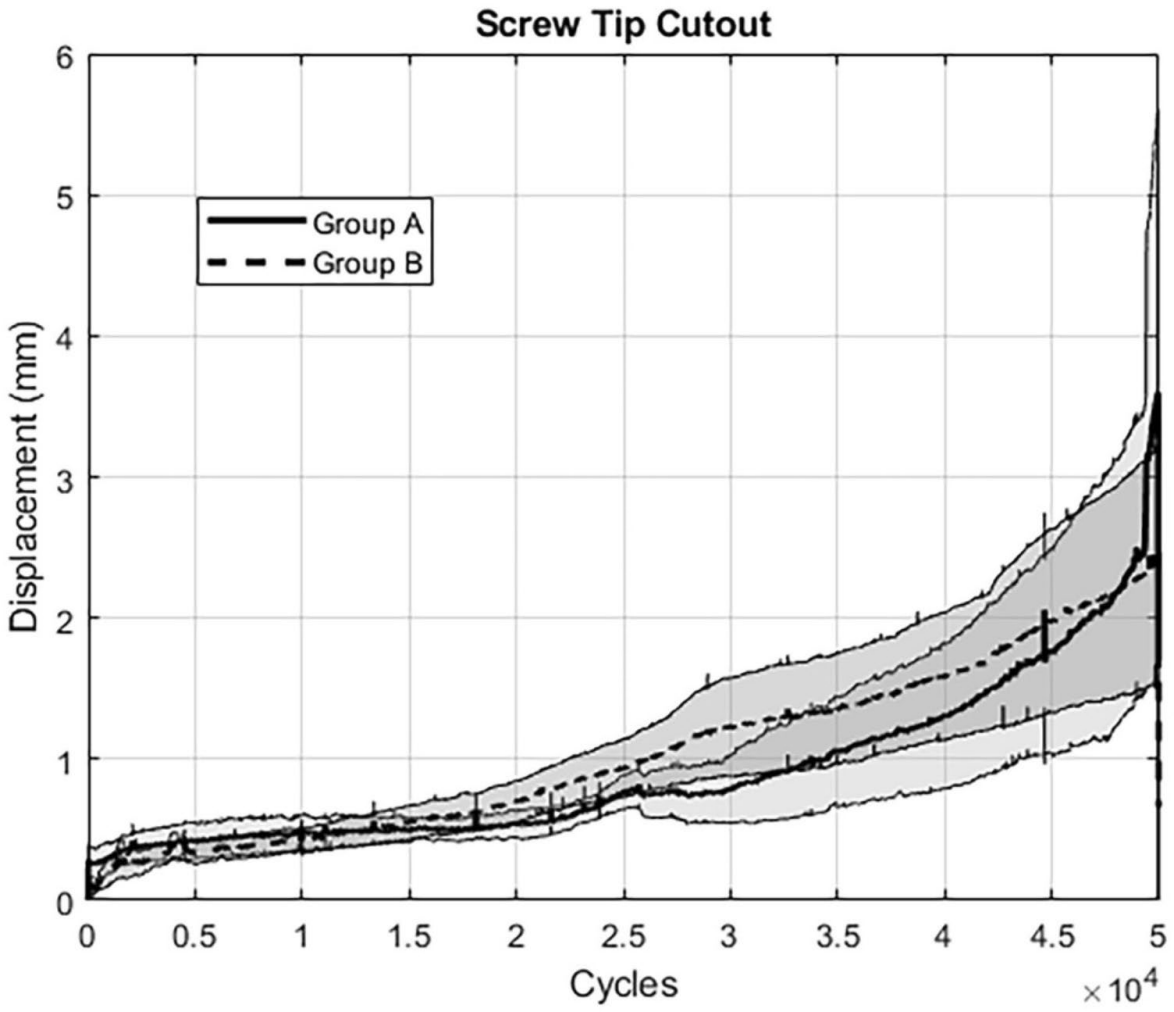
Graph showing results for the assessed parameter “Screw Tip Cutout”: The extent of displacement (in mm) over the course of the first 50.000 cycles is depicted for both groups separately (mean ± SEM)

All outcome measures analyzed over the five time points after 10,000, 20,000, 30,000, 40,000, and 50,000 cycles are summarized in Table 1. Complementary continuous values over the first 50,000 cycles are shown for (i) “gap angle” (Fig. 5), (ii) “screw tilt ilium” (Fig. 6), and (iii) “screw tip cutout” (Fig. 7). For the pooled data sets over the five time points, relative interfragmentary movements in terms of “gap angle” were associated with significantly higher values for group A (screw tip augmentation; median [CI] 2.4° [2.3, 4.9]) versus group B (midshaft augmentation; median [CI] 1.4° [1.0, 1.6]); *p* < 0.001). Similarly, the pooled outcome measure “screw tilt” was associated with significantly higher values following instrumentation of group A (median [CI] 3.3° [3.0, 8.9]) versus group B (median [CI] 1.4° [1.2; 2.9]; *p* = 0.003). However, for “screw tip cutout” no significant difference was obtained between group A (median [CI] 0.6 mm [0.6, 1.3]) and group B (median [CI] 0.8 mm [0.8, 1.8]; *p* = 0.376). Finally, comparison of the reproducibility revealed significantly higher pooled SD's for group A (median [CI] 1.2 [0.9, 4.6]) versus group B (median [CI] 0.5 [0.4, 1.4]; *p* = 0.017).

**Table 1:** Outcome measures of the parameters of interest evaluated after 10,000, 20,000, 30,000, 40,000, and 50,000 cycles, presented for each treatment group in terms of mean value and (standard error of the mean, SEM), with *p*-values indicating differences between the groups

Parameter	Group	Cycles					*p*-value
		10,000	20,000	30,000	40,000	50,000	
Gap angle	A	1.06 (0.33)	1.56 (0.36)	4.17 (1.16)	5.76 (1.59)	5.90 (1.85)	< 0.001
	B	0.66 (0.22)	0.87 (0.19)	1.18 (0.07)	1.54 (0.09)	2.34 (0.21)	
Screw tilt ilium	A	1.27 (0.22)	2.22 (0.44)	6.72 (2.41)	9.75 (3.76)	10.69 (5.74)	< 0.001
	B	0.69 (0.21)	1.18 (0.31)	1.85 (0.58)	2.60 (1.00)	3.86 (1.40)	
Screw tip cutout	A	0.44 (0.12)	0.49 (0.11)	0.80 (0.24)	1.29 (0.51)	1.74 (0.62)	0.376
	B	0.40 (0.11)	0.67 (0.17)	1.20 (0.37)	1.59 (0.48)	2.39 (0.87)

## Discussion

Pelvic (fragility) fractures resulting from low-energy trauma are increasingly frequently observed in patients older than 60 years of age [33]. Moreover, the incidence of pelvic fractures in elderly people (> 80 years) rose from 73 to 364/100.000 individuals from 1970 to 2013 [34]. Consequently, a 127% increase of the total number of hospitalizations following pelvic fractures in older patients (≥ 65 years) between 1986 and 2011 has been reported [35]. A 2.4 fold increase for the occurrence of fractures is predicted for the year 2030 [34] making fragility fractures of the pelvis an increasingly important public health care issue and economic burden [36].

Fragility fractures of the pelvis with moderate instability (FFP IIB) account for the majority of these injuries and surgical treatment with sacroiliac screws is the clinical practice. However, failure rates of sacroiliac screws occurred in 14% to 17% of patients [37, 38]. Accordingly, cement augmentation of sacroiliac screws at the level of the body of S1 vertebra was proposed to increase the anchorage between the bone and the implant and was a matter of preclinical research [15–20]. In addition, a 25% reduced time from surgery to discharge was noted following cement augmentation [23]. However, there are still some controversies and open questions in the existing literature with regard to the efficiency of cement augmented sacroiliac screws [21, 22].

To our knowledge, the site for cement augmentation of sacroiliac screws in FFP IIB fractures providing the highest construct stability has not been defined clearly yet. We hypothesized that cement augmentation of sacroiliac screws at the weakest part (lateral mass of S1) enhances construct stability compared to current practice (body of S1). The rationale for that hypothesis and the presented study is given by previous consistent findings as follows: The lowest bone quality was noted in the lateral portions of S1 which contain yellow marrow and an alar void, associated with minimal regional bone density [24, 39–41]. To that effect, sacral stress fractures occur at this site if the applied shear stresses exceed the mechanical strength of the sacral ala, e.g. due to an increased strain caused by osteoporotic defects in this region [42, 43]. Therefore, medial cement augmentation (vertebral body of S1) at the tip of the screw will not stabilize the lateral alar void defect.

The presented biomechanical analysis with cyclic loading demonstrates that augmentation of the lateral mass compared to the one in the center of the vertebral body of S1 results in less angular displacement between the sacral fragments medial and lateral to the fracture, as well as between the screw and the pelvic bone itself. In summary, the screws with a cement-augmented lateral mass provided a construct being more stable under cyclic loading.

The results confirm the hypothesis of the study, as the cementation at the weakest part of the sacrum enhanced stability. This finding might parallel the noted so-called “windshield-wiper effect” in reconstruction surgery of the anterior cruciate ligament (ACL) as follows: Transverse motion of the ACL graft in the proximal tibial bone tunnel results in widening of the osseous tunnel at risk for failure of ACL reconstruction surgery [44, 45]. Accordingly, the cement augmentation at the tip of the sacroiliac screw might not reduce the long lever arm of the screw with its poor anchorage in the sacral alar. Thus, transverse motion might result in loosening of the screw with backing out. This mechanism might be a potential explanation for the failure of the sacroiliac screw, however, needs to be assessed in other biomechanical experiments. Finally, the technique of cement augmentation at the region “from-screw-head-to-midshaft” rather than at the level of the screw tip has been introduced successfully for management of odontoid fractures in osteoporotic bone earlier [46].

Another explanation might be that the osteosynthesis in these pathologic fractures acts comparably with a compound osteosynthesis [47]. Using this technique, the healing of the pathological fracture is rather not the main purpose (although possible) of the treatment than the durability of the osteosynthesis providing long term stability. The management of FFP in osteoporotic fractures might be comparable as fracture healing is uncertain and the main treatment goals are prevention from fracture progression and the so-called “fracture disease” [12, 48].

A limitation of the study might be concerns about the clinical application of cement injection at the sacral lateral mass using fully threaded screws with the potential risk for cement leakage in the fracture gap and/or no compression at the fracture site preventing fracture healing. However, the following arguments might abolish these concerns: (1) Cement leakages are observed (e.g. as “retrograde” leakage) regardless of the site of cement application [21], (2) surgeons rather should be aware of the physical properties of the cement and the “bone permeability”[49, 50] and using a balloon guided cement augmentation was successful in leakage prevention [51], (3) using sacroplasty for sacral insufficiency fractures appears to be safe and efficient in current available literature, however, controlled randomized clinical trials are lacking [52], (4) the need and the surgical feasibility to obtain compression at the fracture site is questionable as (i) the FFP IIB fracture is a result of (already occurred) lateral compression, (ii) from a surgeon`s perspective there is the risk for stripping of the screw or cut-through of the washer through the weak innominate bone, (iii) a recent biomechanical study did not reveal any differences in four out of nine cadavers in a compression test analyzing fully versus partially threaded screws even in rather young (mean age 68 years), male donors (atypical patients in terms of FFP) whereas an overall significant difference of 1 mm in fracture site compression (questionable benefit versus risk for screw stripping) was reported in favor of partially threaded screws [53], (5) cement augmentation at the weakest site with filling the void is a technique known as “compound osteosynthesis” in pathological fractures of extremities in patients with limited life expectancies without claiming for fracture healing [47]. In summary, there are many arguments to repeal these concerns above. Another limitation might be the low number of specimens and the biomechanical setup per se. However, there is a limited availability of fresh-frozen human cadavers; the experimental setup was designed to detect differences between two study groups to provide evidence for a design modification and the rationale to reconsider current clinical practice. Potential further limitations of the present study arise from the nature of the biomechanical protocol, e. g. the resection of the symphysis does not reflect clinical reality. However, the rationale to resect the symphysis was to obtain a reproducible setup for both sides allowing for internal rotation of both hemipelvises in the horizontal plane. For placement of the sacroiliac screw no insertion guide was used. However, CT scans were performed after screw placement and ruled out any relevant side-to-side differences in screw positioning and further confirmed correct intraosseous screw placement. In addition, we developed a preoperative planning approach for sacroiliac screw placement [54], which supports reproducible screw positioning and procedures were performed by a senior consultant surgeon experienced with this technique.

The strengths of the study are: (1) we used fresh-frozen human cadavers to reflect the clinical situation rather than synthetic bone models without any ligaments attached to the bone, (2) to ensure homogeneity and comparability of these specimens clear inclusion criteria were defined and analyzed using CT scans; including a phantom to detect differences in bone mineral density, (3) to allow for direct comparison between the two groups, both techniques were tested within the same bone quality within one cadaver, (4) a FFP IIB fracture was tested by generating “a posterior undisplaced sacral crush injury with an anterior ring disruption” to reflect the most frequent fracture pattern, (5) for fixation, we used fully threaded screws as these provided more stability in osteoporotic bone by improved anchorage and by fixation within the cortical bone of the sacroiliac joint; accordingly, we obtained a high fixation strenght at baseline before application of cement, (6) all osteosynthetic constructs were tested under cyclic loading to reflect the physiological loading as much as possible with shear stresses concentrated at the sacral lateral mass.

## Conclusion

The presented study demonstrates less fragment and screw displacement in a FFP IIB fracture model under physiologic, cyclic loading by cement augmentation of sacroiliac screws at the level of the lateral mass compared to the center of vertebral body of S1 for the first time. The study results provide the rationale to assess the potential benefit of this technique in a clinical setting.

## Data Availability

The data required to reproduce the above findings cannot be shared.
